# Biological and Molecular Characterization of the Lytic Bacteriophage SoKa against *Pseudomonas syringae* pv. *syringae*, Causal Agent of Citrus Blast and Black Pit in Tunisia

**DOI:** 10.3390/v14091949

**Published:** 2022-09-02

**Authors:** Maroua Oueslati, Dominique Holtappels, Kiandro Fortuna, Mohamed Rabeh Hajlaoui, Rob Lavigne, Najla Sadfi-Zouaoui, Jeroen Wagemans

**Affiliations:** 1 Laboratoire de Mycologie, Pathologies et Biomarqueurs (LR16ES05), Département de Biologie, Université de Tunis-El Manar, Tunis 2092, Tunisia; 2 Laboratory of Gene Technology, Department of Biosystems, KU Leuven, 3001 Leuven, Belgium; 3 Laboratoire de Biotechnologie Appliquée à l’Agriculture, INRA Tunisia, Université de Carthage, Ariana 2094, Tunisia

**Keywords:** phage biocontrol, citrus, bacteriophage, fruit bioassay

## Abstract

*Pseudomonas syringae* pv. *syringae* (Pss), the causal agent of citrus blast and black pit lesion of lemon fruit, continues to cause serious damage in citrus production in Tunisia. Faced with the rapid emergence of the disease and the inefficiency of conventional control methods, an alternative strategy based on the use of bacteriophages was pursued in this study. The lytic Pss bacteriophage SoKa was isolated from soil collected from Tunisian citrus orchards. Analysis of the host range showed that SoKa was able to lyse seven other Pss strains. Interestingly, *Pseudomonas syringae* pv. *porri*, pathogenic to leek, could also be infected by SoKa. The activity of SoKa was maintained at pH values between 2 and 10, at temperatures between −80 and 37 °C; the phage could resist UV radiation at an intensity of 320 nm up to 40 min. Whole genome sequencing revealed that the *Pseudomonas* phage SoKa is a novel phage that belongs to the *Bifseptvirus* genus of the *Autographiviridae* family. The absence of virulence proteins and lysogeny-associated proteins encoded on the phage genome, its anti-biofilm activity, and the significant reduction of tissue necrosis in different fruit bioassays make SoKa potentially suitable for use in phage biocontrol.

## 1. Introduction

Phytopathogenic *Pseudomonas syringae* has two interconnected growth phases. First, an epiphytic phase, occurring mainly in autumn and winter, during which the bacteria colonize the phylloplane of citrus hosts and form a biofilm. Second, an endophytic phase in spring and summer, in which the biofilm degrades, followed by the migration and penetration of bacterial cells through the stomata and micro-injuries in the plant. This is followed by the release of toxins and plant cell-degrading enzymes, thus causing the appearance of typical disease symptoms on fruits, leaves, and twigs [1]. Chemical measures to control fruit tree diseases caused by *P. syringae* pv. *syringae* are limited and often ineffective. To minimize bacterial infections, several measures have been proposed, such as the use of pathogen-free seeds, resistant cultivars, and appropriate agricultural practices [2]; however, these are also not effective enough. Primarily, the endophytic nature of this pathogen combined with the consumer’s preference for ecological products and government regulations have prompted researchers to study the potential of phage biocontrol as an adjunct control strategy [3].

Bacteriophages developed for the control of plant pathogenic bacteria exploit the complex virus–host interaction to considerably reduce disease symptoms, economic losses, and to minimize the impact on the environment and on non-target microorganisms [4,5]. Thus, phages are considered to be more sustainable and safer than antibiotics because they are naturally present in the environment. By contrast, most of the commonly used chemical products are non-specific and also target commensal bacteria that are naturally present in the plant microflora. Conversely, phages are generally highly specialized. This feature is beneficial as it reduces the impact on the host’s bacterial community, but it constitutes an obstacle to eradicate all disease-causing individuals of the community [6]. Hence, there is usually a need to apply several types of phages to cover the diversity of the target species or pathovars [7].

Besides the lack of a specific regulatory framework, several other limitations hinder the successful application of phages in agriculture. Most phages are sensitive to UV radiation, which causes a rapid decay in foliar applications [8]. Timing and adjusting the precise moment of application in anticipation of the pathogen are therefore of great importance to improve the success of phage applications in general [9]. In addition, protective formulations using substances such as natural extracts of carrot, red pepper, and beetroot as well as casein, soy peptone, amino acids, Tween 80, and skim milk can increase the survival of phages on the plant surface [10]. Some other methods have been proposed to solve this persistence issue. For instance, phage viability can be improved in both the phyllo- and the rhizosphere if accompanied by a compatible non-pathogenic host, such as an avirulent strain or another susceptible species naturally present in similar environments [11]. 

Another hurdle phage biocontrol needs to overcome is resistance development. Just as bacteria can become resistant to antibiotics, they can also become resistant to phages by various mechanisms. These include the modification of phage surface receptors on the bacterial cell via physical barriers or via the production of competitive inhibitors [12,13], the blocking of the entry of phage DNA, modification of the phage DNA [14], or interruption of the infectious cycle (abortive infection systems). To prevent the development of bacterial resistance to phages, the standard practice is to use mixtures or ‘cocktails’ which may contain combinations of phages with narrow and broad host ranges [15].

Several lytic phages have been described for *P. syringae* pathovars, including *P. syringae* pv. *tomato* [16], *P. syringae* pv. *phaseolicola* [17,18], *P. syringae* pv. *porri* [9], *P. syringae* pv. *actinidiae* [18,19,20], *P. syringae* pv. *aesculi* [21], and *P. syringae* pv. *syringae* [22]. Although some studies have tested the efficacy of certain phages against the *P. syringae* complex, no study has so far reported on the isolation and characterization of phages with potential lytic activity against the citrus phytopathogenic *Pseudomonas,* and particularly against *P. syringae* pv. *syringae* PG02b, the most aggressive phylogroup in Tunisian citrus orchards [23,24]. In the present study, we report on the isolation and molecular characterization of the SoKa phage.

## 2. Materials and Methods

### 2.1. Isolation, Purification, and Amplification of Bacteriophages

Phages were isolated from soil, irrigation water, and symptomatic lemon samples from citrus orchards that were known to be previously infested with *P. syringae* pv. *syringae* [23,25]. To enrich samples for the phages present, the host bacterium (KB49), previously isolated from citrus black pit, was cultured in 30 mL of low-salt (0.5 g/L) lysogeny broth (LB_ls_) and incubated at 25 °C until the early exponential growth phase (optical density (OD) of 0.3 at 600 nm). A total of 10 g of soil sample was added. After overnight incubation, this mixture was centrifuged (30 min, 4000 rpm), and the supernatant was filtered through a membrane filter with a pore size of 0.45 µm. A 10 µL aliquot of the filtrate was deposited onto a layer of LB_ls_ soft agar (0.5% agar) mixed with 200 μL of an overnight culture (OD 0.8–1). After overnight incubation at 25 °C, lysis zones were picked with sterile toothpicks and suspended in phage buffer (10 mM Tris-HCl; pH 7.5; 10 mM MgSO_4_; 150 mM NaCl). The phages were then purified using the same agar overlay method [26] by mixing 250 µL of a bacterial suspension with 100 µL of the phage suspension and 4 mL of LB_ls_ soft agar and then spreading it on an agar plate. After overnight incubation at 25 °C, individual plaques were picked again. Three successive isolations of a single plaque were performed to obtain pure phage isolates. Subsequently, the pure isolates were amplified by plating 10^5^ plaque-forming units (PFU) with their bacterial host. The next day, the soft agar was scraped off and suspended in phage buffer. The soft agar and cell debris were then removed by centrifugation (30 min, 4000 rpm) and filtration (0.45 μm). Next, cold polyethylene glycol (PEG) 8000 (Sigma-Aldrich; dissolved in 1 M NaCl at a stock concentration of 25% *w*/*v*) was added to the suspension at a final concentration of 8% *w*/*v*. After overnight incubation at 4 °C with continuous shaking, the phages were precipitated by centrifugation (30 min, 4000 rpm), and the pellet was resuspended in 1 mL of phage buffer [9].

### 2.2. Microbiological Characterization of the Phage

#### 2.2.1. Determination of the Host Range

The host range of the SoKa phage was evaluated on *Pseudomonas* spp. and other phytopathogenic bacteria: *Dickeya* and *Agrobacterium* (Table 1). The tested *P. syringae* pv. *syringae* strains were all isolated from symptomatic citrus in Tunisia between 2015 and 2017. In addition to the pathovar *syringae*, we also tested *P. syringae* pv. *porri* (Table 1). The host spectrum was determined by a spot assay. Bacteria were cultivated in an LB_ls_ to an OD of 0.5 and for each tested strain, 200 μL was plated using the agar overlay method. Once the overlay had solidified, 10 μL of a dilution series of the phage (up to approximately 10^5^ PFU/mL) was spotted on the agar. Plates were incubated at 25 °C and examined after 24 h. Positive results (showing lysis zones) were confirmed by plating the dilution series (100 µL of each dilution) using the double agar layer method. Bacterial susceptibility was evaluated by checking for plaque formation and bacteria were categorized as resistant or susceptible.

#### 2.2.2. Electron Microscopy

Transmission electron microscope (TEM) images of the isolated bacteriophage were obtained as described previously [18].

#### 2.2.3. One-Step Growth Curve

The host bacterium was incubated at 25 °C until mid-exponential phase (OD 0.3, corresponding to about 1.25 × 10^8^ CFU/mL). The suspension was infected with 10^6^ PFU (MOI = 0.01). Phages were allowed to adsorb for 10 min (25 °C) and the mixture was centrifuged twice at 14,000× *g* for 1 min to remove the non-adsorbed phages. The pellet was then suspended in 1 mL of LB_ls_, diluted to 10^−3^ in LB_ls_, and incubated at 25 °C with shaking (100 rpm). Every five or ten minutes, 100 μL were taken and the phage titer was determined immediately using the agar overlay method. All tests were carried out in triplicate and incubated overnight at 25 °C. The latent period is the time interval between infection and the start of phage production. The burst size was calculated as the ratio of the final number of phage particles released to the initial number of infected bacterial cells during the latency period [27].

#### 2.2.4. Influence of pH on Phage Viability

Phages (10^7^ PFU/mL) were suspended in 1 mL of phage buffer, previously adjusted with 1 M NaOH or 1 M HCl, to have a pH range of 1 to 13. After 24 h of incubation at 25 °C, serial dilutions were prepared. Each sample was tested against the host bacterium to verify phage viability. Analyses were carried out in triplicate and results were reported as the average of the number of lysis plaques (PFU/mL).

#### 2.2.5. Effect of Temperature on Phage Viability

The phage particles (10^7^ PFU mL^−1^) were suspended in 1 mL of phage buffer and incubated at −80 °C, −20 °C, 4 °C, 25 °C (control) and 37 °C for 24 h. For the highest temperatures, 50 °C, 80 °C and 100 °C, the solutions were incubated for 4, 8 and 24 h. The surviving phages were diluted in series, and then plaques were counted. Each experiment was carried out three times and the average of the phage titer (PFU/mL) and the standard deviation were determined.

#### 2.2.6. Effect of UV on Phage Viability

To evaluate the effect of UV irradiation (290–320 nm), a type B ultraviolet lamp was used. Phage samples (10^7^ PFU/mL) were placed directly on the TL 20 W/12 RS lamp (Philips, Holland). The experiments were carried out in phage buffer at pH 7.0 and at room temperature. Samples were exposed for 10, 20, 30, 40, 60 or120 min and serial dilutions were prepared. They were plated in triplicate (technical repeats) for incubation at 25 °C. The average number of lysis ranges and the standard deviation were calculated.

### 2.3. Molecular Characterization of the Phage

#### Genome Analysis

Free bacterial DNA and RNA were removed from the phages via the treatment of a 1 mL stock with 10 µg DNase I and 50 µg RNase A at 37 °C for 30 min. To inactivate enzymes and destroy the phage particles, 40 µL of 0.5 M EDTA was subsequently added, together with 50 µL of SDS (10%) and 10 µL of proteinase K (50 µg/mL), followed by incubation in a water bath at 56 °C for 1 h. The released DNA was purified by phenol-chloroform extraction. DNA concentration and purity were assessed with a NanoDrop ND1000 spectrophotometer (Thermo Fisher Scientific Inc., Waltham, MA, USA). The integrity and the size of the genome were evaluated on a 0.8% agarose gel. 

Whole genome sequencing was performed using an in-house Illumina MiniSeq platform (Illumina, San Diego, CA, USA), according to instructions described by Makalatia et al. [28]. After genome assembly, the most related phages were identified with BLASTn [29] and Viptree v1.9 [30]. VIRIDIC [31] was used for taxonomic classification, while the heatmap was created with ggplot2. Next, annotation was performed with RASTtk [32], followed by manual curation using BLASTp. The lifecycle was predicted using the PhageAI platform [33] and further confirmed by the absence of integrase genes in the genome. Potential virulence and antibiotic resistance genes were identified using VirulenceFinder [34] and ABRicate [35], respectively. The packaging strategy of SoKa was determined based on the work of Merrill et al. [36], namely by comparing the large subunit of the terminase protein to terminases with known packaging systems. A genome map was visualized with Easyfig [37]. The data were submitted to NCBI GenBank under accession number OM962999.

### 2.4. Effect of Bacteriophage on Biofilm Formation

#### 2.4.1. Glass Slide Method

To highlight the anti-biofilm potential of phages, biofilms were grown on glass slides, as described by Shafique et al. [38]. The glass slides were dipped into Petri dishes containing 20 mL of sterile LB broth, bacterial suspension (2 × 10^8^ CFU/mL), and phage lysate (4 × 10^8^ PFU/mL). After a 72 h incubation, the glass slides were removed from the plates and rinsed three times with sterile distilled water to remove loosely attached cells. The slides were air-dried, covered with 0.1% crystal violet for 5 min, and then washed three times with 0.85% normal saline (NaCl). Next, the slides were air-dried again, and biofilm formation in the presence/absence of phages was observed and evaluated under light microscopy. The same procedure was carried out on a negative control slide dipped into LB broth only, and on a positive control slide that had a bacterial biofilm with no phage exposure; this allowed for a comparison of the amount of biofilm formed on the slides treated and non-treated with phages. The experiment was repeated three times.

**Table 1:** Host Range of the SoKa phage on different host bacteria. A plus or minus sign respectively indicates susceptibility or resistance to phage infection. Culture collections: NCPPB—National Collection of Plant Pathogenic Bacteria, Harpenden, UK; MAFF—Ministry of Agriculture, Forestry, and Fisheries, Tsukuba, Ibaraki, Japan; CFBP—Collection Française des Bactéries Phytopathogènes, Institut National de la Recherche Agronomique, Beaucouzé Cedex, France.

Species and Pathovars	Strains	Plant Hosts	Infectivity	References
*Pseudomonas syringae* pv. *syringae*	E11	Citrus	−	[23]
*Pseudomonas syringae* pv. *syringae*	EL1A	Citrus	−	[23]
*Pseudomonas syringae* pv. *syringae*	BE1	Citrus	−	[23]
*Pseudomonas syringae* pv. *syringae*	BE3	Citrus	−	[23]
*Pseudomonas syringae* pv. *syringae*	KC19	Citrus	+	[23]
*Pseudomonas syringae* pv. *syringae*	KC46	Citrus	+	[23]
*Pseudomonas syringae* pv. *syringae*	KC82	Citrus	+	[23]
*Pseudomonas syringae* pv. *syringae*	TRR12	Citrus	+	[23]
*Pseudomonas syringae* pv. *syringae*	TRR9	Citrus	+	[23]
*Pseudomonas syringae* pv. *syringae*	E9A	Citrus	−	[23]
*Pseudomonas syringae* pv. *syringae*	E12A	Citrus	+	[23]
*Pseudomonas syringae* pv. *syringae*	IyGC	Citrus	+	[23]
*Pseudomonas congelans*	BE12A	Citrus	−	[23]
*Pseudomonas nabeulensis*	E10AB	Citrus	−	[25]
*Pseudomonas kairouanensis*	KC20	Citrus	−	[25]
*Pseudomonas savastanoi* pv. *savastanoi*	2C	Olive	−	unpublished
*Pseudomonas savastanoi* pv. *savastanoi*	2E	Olive	−	unpublished
*Agrobacterium rhizogenes*	NCPPB 4042	Cucumber	−	NCPPB
*Agrobacterium rhizogenes*	NCPPB 2659	Cucumber	−	NCPPB
*Agrobacterium tumefaciens*	MAFF 210265	Melon	−	MAFF
*Dickeya dadantii*	NCPPB 3537	Potato	−	NCPPB
*Dickeya chrysanthemi*	NCPPB 402	Chrysenthemum	−	NCPPB
*Pseudomonas syringae* pv. *porri*	CFBP 1770	Leek	+	CFBP
*Pseudomonas syringae* pv. *porri*	GBBC 1269	Leek	+	[39]
*Pseudomonas syringae* pv. *porri*	GBBC 3224	Leek	+	[39]
*Pseudomonas syringae* pv. *porri*	Pspo 1277	Leek	+	[39]
*Pseudomonas syringae* pv. *porri*	P55	Leek	+	[40]

#### 2.4.2. Microplate Method

The ability of the phage to inhibit biofilm formation on polystyrene microplates was determined by crystal violet staining. Ten microliters of phage suspension (10^7^, 10^8^, and 10^9^ PFU/mL) were mixed with a bacterial suspension in the exponential phase (10 μL), in microplate wells of 200 µL LB_ls_, and then incubated at 28 °C for 72 h. Wells inoculated with the bacteria were used as a positive control, and the LB medium was used as a negative control. For each condition, six replicates were included. After incubation, the wells were emptied and rinsed three times with saline solution (0.85%) to remove planktonic cells and then filled with 200 μL of crystal violet (0.4% in methanol) to stain the biofilm formed in the wells. After 20 min, the wells were rinsed three times with sterile distilled water, and 200 µL of 33% acetic acid was added to dissolve the dye. Optical density was then measured at 580 nm.

### 2.5. Bioassay on Detached Fruits

Lemons (*Citrus limon* cv. Eureka) were purchased from a local supermarket. Fruits were washed with distilled water, disinfected in 1% HClO (hypochlorous acid) for 5 min, rinsed with sterile water and washed with 70% ethanol, and allowed to dry at room temperature under a laminar flow hood. Tests were carried out under five conditions, and for each test, six fruits were inoculated. A phage solution (100 µL, 10^8^ PFU/mL) was injected into the fruit. After one hour, the same volume of the bacterial suspension (OD 0.1) was added using the same injection point. Fruits inoculated with bacterium and phage buffer were used as positive control. Negative controls were treated with (1) phage only and growth medium, (2) inactivated phage (by UV treatment for 2 h) and medium, and (3) medium and phage buffer. Symptoms were scored by measuring the diameter of the necrosis zones relative to the total fruit diameter. In a second bioassay, the same experiment was repeated on lemons, grapefruits (*Citrus* × *paradisi*), and oranges (*Citrus × sinensis*). Here, the phage-only negative control was not considered. The percentage of necrotic tissue (weight of dead tissue compared to total weight) was measured.

### 2.6. Statistical Data Analysis

Statistical analyses for the antibiofilm assay and the fruit bioassays were performed with JMP Pro 16 (SAS, Cary, NC, USA). For all analyses, a confidence level of 0.05 was applied. Normality of data was assessed with a Shapiro–Wilk test, while equality of variances was verified using an O’Brien test (for non-normally distributed data sets) or a Bartlett test (for normally distributed data sets). When the variance was equal, a one-way ANOVA was run with a Student’s *t*-test to compare the means of the different treatment groups with the positive control in the case of normal data, while the Games–Howell test (a Tukey HSD post hoc analysis assuming unequal variances) was used for non-normal data. For unequal variances, the Wilcoxon Each Pair test was calculated.

## 3. Results

### 3.1. Isolation and Microbiological Characterization of the P. syringae Podovirus SoKa

**Figure 1:**
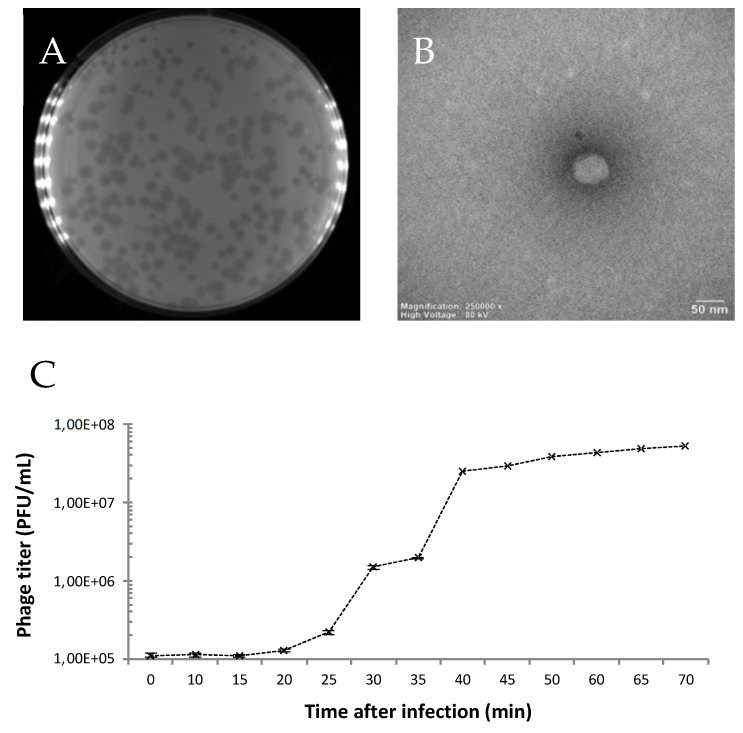
Morphology and one-step growth curve of SoKa. (**A**) Macroscopic view of the SoKa plaques on a bacterial lawn shows clear lysis plaques with a diameter of 0.5–2 mm. (**B**) TEM micrograph of SoKa displays a podovirus morphology with a head diameter of 60 nm. Bar represents 50 nm. (**C**) One-step growth curve of SoKa on the *Pseudomonas syringae* pv. *syringae* strain KB49 at different time points. Each data point is a mean from three experiments. The error bars show the standard error of the mean.

The phage ‘SoKa’ was isolated from soil taken from a citrus orchard in the Kairouan region in Tunisia. Soil (11), irrigation water (3), and symptomatic lemon (2) samples were assessed for the presence of phages active against the citrus phytopathogenic *P. syringae* pv. *syringae*. A single sample of these 16 contained a culturable phage. From the remaining samples, no phages could be isolated (Table 1). SoKa formed clear lysis plaques with a diameter of 0.5–2 mm (Figure 1A) on its isolation strain KB49, which had previously been isolated from the same region [23]. The morphology of the SoKa virion was studied by TEM. The phage has a typical podovirus morphology, consisting of an icosahedral head of 60 nm with a very short, thin, and non-contractile tail (Figure 1B). The phage has a latency period of 20 min and a burst size of 49 infectious phage particles per cell (Figure 1C). 

A host range analysis showed that SoKa was able to infect eight other *P. syringae* pv. *syringae* strains (KC19, KC46, KC82, TRR12, TRR9, E12A, and IyGC) out of 12 tested strains, all belonging to the PG02b phylogroup [23]. Interestingly, all five tested strains of *P. syringae* pv. *porri* were also susceptible. No other *Pseudomonas* species nor other tested genera (*Agrobacterium, Dickeya*) were susceptible to SoKa infection (Table 1).

**Figure 2:**
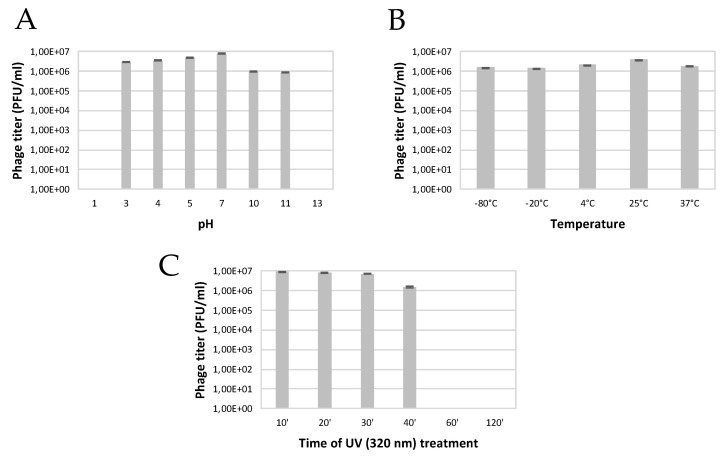
Stability of SoKa under different environmental conditions. (**A**) Effects of pH changes. (**B**) Activity of SoKa at different temperatures. (**C**) Effect of UV treatment on the stability of the SoKa phage. The error bars show the standard error of the mean.

The SoKa phage was stable in a wide range of pH values as it only completely lost its infectivity after 24 h at pH 1 and pH 13, while remaining infective between pH 2 and 10, with an optimum at around pH 7 (Figure 2). The effect of temperature on phage viability was assayed at different temperatures ranging from −80 °C up to 100 °C. Results showed that the phage particles of SoKa are viable after being incubated from −80 °C until 37 °C for 24 h. After four hours of incubation at 80 °C and 100 °C, no more plaques were obtained. Treatment at 50 °C quickly reduced infectivity after eight hours of incubation and completely inactivated the phage after 24 h. The effect of UV exposure showed that SoKa could resist up to 40 min at an intensity of 320 nm. A loss of phage viability was recorded after 60 min.

### 3.2. SoKa Is a Proposed New Species within the Bifseptvirus Genus of the Autographiviridae Family

**Figure 3:**
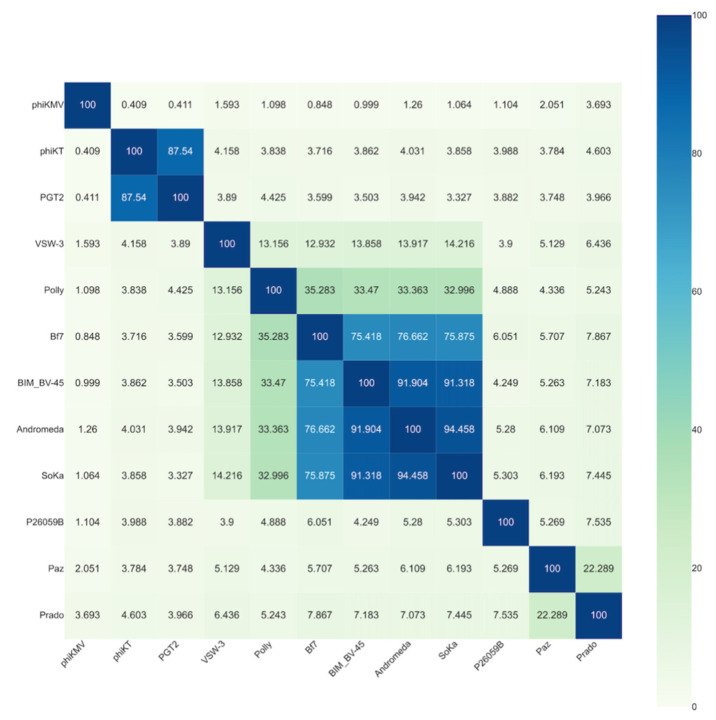
VIRIDIC (Virus Intergenomic Distance Calculator) heatmap comparing the phages most related to SoKa. The legend shows the intergenomic similarity between each pair of phages as a percentage of sequence identity. Phages sharing more than 70% identity are members of the same genus, thus showing that SoKa belongs to the same genus as Bf7, BIM BV-45 and Andromeda.

The SoKa genome was determined using whole genome sequencing. This revealed a dsDNA genome of 40,360 bp, with BLASTn similarity to *Pseudomonas* phages BIM BV-45 (MT094430; 96% coverage; 94.67% identity), Andromeda [41] (KX458241; 97% coverage; 96.76% identity), and Bf7 [42] (JN991020; 86% coverage; 83.22% identity). To classify SoKa, the intergenomic distance to related bacteriophages (identified with a Viptree proteome analysis) was calculated and plotted (Figure 3). This revealed that the *Pseudomonas* phage SoKa is a novel phage species belonging to the *Bifseptvirus* genus of the *Autographiviridae* family.

**Figure 4:**
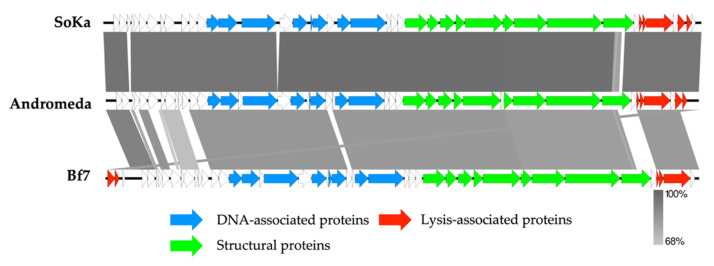
Genome map of the sequenced Pseudomonas phage SoKa and comparison (greyscale) to the closest related phages, Andromeda and Bf7, using BLASTn analysis. Each arrow represents a coding sequence. In red—genes encoding packaging and lysis-associated proteins are displayed; in green—structural proteins; in blue—DNA- and metabolism-associated proteins (adapted from EasyFig).

The SoKa genome was realigned to the Andromeda genome and then annotated. Structural annotation identified 42 coding sequences and no tRNAs. Twenty-two coding sequences could be assigned a putative function (Figure 4). Similar to other *Autographiviridae* phages, which are characterized by the presence of a phage DNA-directed RNA polymerase, the genome can clearly be divided in the early gene module, followed by the replication module, the structural genes cassette, and finally, the lysis-related genes. The large subunit of the terminase was compared to similar proteins with known packaging systems using the method described by Merrill et al. [36], which indicated that SoKa uses short direct terminal repeats (DTRs) for packaging its DNA inside the phage particles.

No genes encoding lysogeny-associated proteins were identified, which was confirmed by the Phage.AI lifecycle predictor. Moreover, no known virulence factors nor antibiotic resistance proteins were encoded on the phage genome, making it potentially suitable for biocontrol purposes. 

### 3.3. SoKa Displays Significant Antibiofilm Activity

**Figure 5:**
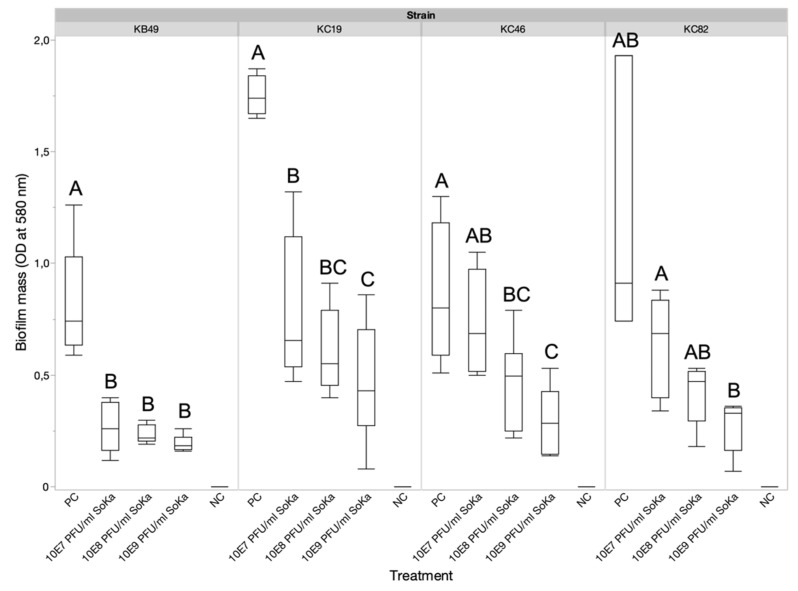
Biofilm mass reductions of different Pss strains by SoKa phage treatment at different concentrations (10^7^, 10^8^, and 10^9^ PFU/mL). For each strain, significant differences between the different treatment groups are indicated by a connecting letters report (*p* < 0.05). For KB49 and KC82 (unequal variances), a Games–Howell test was used, while for the two other strains (equal variances), a Student’s *t*-test was run to compare the means of the different treatments to that of the untreated control. PC = positive bacteria-only control; NC = negative LB medium control.

**Figure A1:**
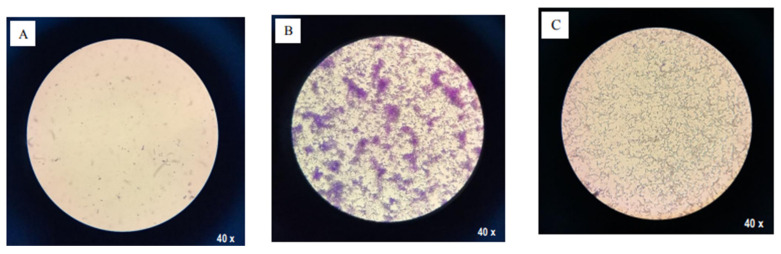
Phage anti-biofilm activity visualized using light microscopy (magnification 40×). (**A**) negative control; (**B**) positive control; (**C**) phage-treated sample. The bacterial biofilms are characterized by the formation of cell cluster structures stained in purple using crystal violet.

To investigate the antibiofilm potential of the SoKa phage, a KB49 biofilm was grown on a glass slide, either with or without the presence of phage (MOI 1). After a 72 h incubation, the biofilm was stained with crystal violet and visualized using light microscopy (Figure A1). By comparing the untreated slide with the one treated with phage, a high reduction in the number of bacterial clusters could be observed due to the phage treatment. To quantify this reduction, a biofilm for each of the SoKa-susceptible Pss strains KB49, KC19, KC46, and KC82 was grown in a microtiter plate for 72 h, either with or without phages and at different concentrations (10^7^, 10^8^, and 10^9^ PFU/mL). The biofilm was stained with crystal violet and the mass was quantified by measuring the optical density at 580 nm. Biofilm formation was significantly inhibited by the presence of the phage as compared to the bacteria-only control in almost all combinations, except for the KC46 strain treated with the lowest concentration of phage and for the KC82 strain (Figure 5). 

### 3.4. A Fruit Bioassay Shows the ex Planta Efficacy of SoKa

**Figure 6:**
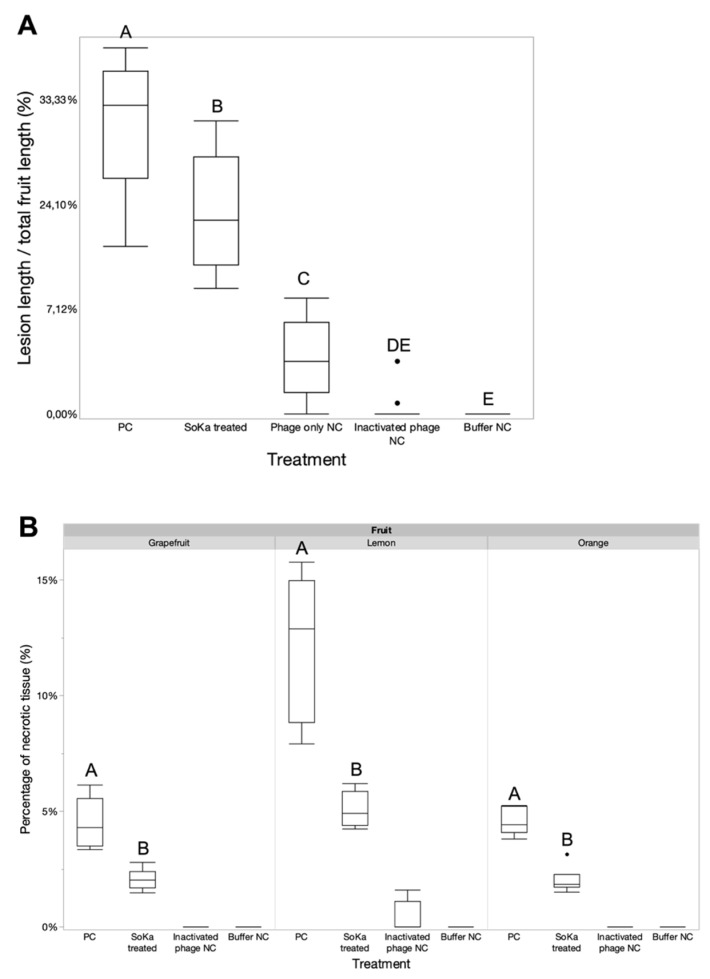
Fruit bioassays to test the efficacy of phage treatment on symptom reduction. (**A**) First phage bioassay on lemon fruits. The lengths of the bacterial lesions induced by strain KB49 were measured and plotted compared to the total length of the fruit. Injection of the SoKa phage significantly reduced the average lesion length compared to control fruits (Wilcoxon test). (**B**) The same bioassay setup was repeated on grapefruit, orange, and lemon fruits. Here, the necrotic tissue of the treated samples were compared with the untreated group (Wilcoxon test for orange, Student’s *t*-test for grapefruit, Games–Howell test for lemon). Significant differences between the different treatment groups (separately for each fruit) are indicated with a different letter (*p* < 0.05). PC = positive bacteria-only control; NC = negative control.

**Figure A2:**
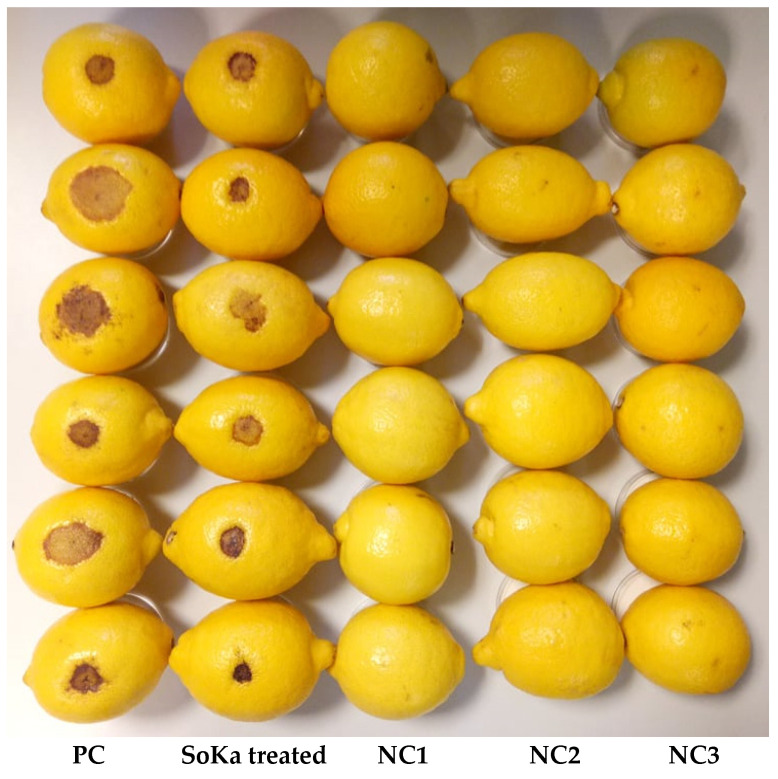
Lemon bioassay to test the efficacy of phage treatment on symptom reduction. Lemons were sterilized, treated with or without phage, and inoculated with the strain KB49. Tests were carried out under five conditions, and for each test, six fruits were inoculated. Fruits inoculated with bacterium and phage buffer were used as positive control (PC). Negative controls (NC) were treated with (1) phage-only and growth medium, (2) medium and phage buffer, and (3) inactivated phage and medium.

In order to evaluate the potential of SoKa to lyse its bacterial host ex planta, a first bioassay was performed on lemon fruits. Bacterial concentration was set at 10^8^ CFU/mL, which is the minimal number of bacteria necessary to produce visible disease symptoms. For the phage, 10^9^ PFU/mL (MOI 10) was used to prove its efficacy. A visual representation of this first experiment is shown in Figure A2. The experiment was repeated, in which the lengths of lesions induced by strain KB49 were measured and plotted (Figure 6A). Injection of the SoKa phage significantly reduced the average lesion length compared to control fruits, from 35.1 to 22.1%. SoKa reduced the symptoms but could not completely prevent bacterial infection. In a second bioassay, the same strategy was repeated on lemons, grapefruits, and oranges, which showed comparable results, with significant reductions in necrotic tissue after phage treatment (Figure 6B). 

## 4. Discussion

*P. syringae* pv. *syringae* is reported to be the most harmful bacterium that causes citrus blast and black pit in Tunisian orchards [23]. Various strategies to control bacterial plant diseases have been explored, including the use of copper products [43]. In addition, biological control using antagonistic bacteria is widely used for the management of bacterial diseases, either by directly stimulating the natural defenses in the host or by directly ensuring the biocontrol of bioaggressors [44]. Currently, the management of most fruit tree diseases caused by Pss is almost impossible due to the lack of effective chemical or biological control measures and the endophytic nature of the pathogen during certain phases of the disease cycle [1]. Therefore, new strategies are needed for the effective management of bacterial diseases. Phage biocontrol was shown to have potential in managing phytopathogenic bacteria [5,22]. This study was conducted with the aim of demonstrating the ability of the SoKa phage isolated from infested citrus orchards to prevent the development of necrosis caused by *P. syringae* pv. *syringae* from phylogroup PG02b.

The SoKa phage was isolated from soil taken from the Kairouan region, and it belongs to the *Bifseptvirus* genus within the *Autographiviridae* family. Based on whole genome sequencing, SoKa was shown to be strictly lytic, thus showing potential for phage biocontrol. In addition, by demonstrating a temperature stability of 4–37 °C and a pH stability of pH 4–10, it can survive in the citrus tree environment after its application. Interestingly, our UV stability assay demonstrated a high tolerance to UV light in lab conditions. As UV light is considered to be one of the main constraints in the application of phages in the plant phylloplane [45], SoKa might be a suitable candidate for biocontrol. However, its efficacy in the field first has to be proven. To reduce resistance development and to control the disease in general (since there are also other opportunistic species of *Pseudomonas* associated with citrus black pit [23,25] besides phylogroup PG02b), however, the current set of phages first needs to be expanded. Another study showed the effectiveness of phage phi6 against *P. syringae* pv. *syringae* in vitro [46]. These results are preliminary but promising findings that encourage other studies to be conducted; further studies could evaluate the potential synergistic effects of SoKa and phi6 in vivo and in planta.

Moreover, if the phages prove their efficacy in field trials, protective formulations should be developed to ensure efficacy in large-scale applications to further increase the stability of the phage. In the literature, diverse formulations are described, including the use of carrier strains, formulations based on skimmed milk, and other natural products [10,47]. Another important factor to ensure a successful outcome in the application is the timing of the application, which has been heavily reviewed by multiple authors [5,47,48]. Additional information on the disease cycle of Pss in citrus is hence inevitably needed to be able to design applications that could prevent an outbreak of the disease in situ [15].

In nature, many bacteria form biofilms to protect themselves from environmental factors. These biofilms enable *P. syringae* pv. *syringae* to colonize plant organs more easily and create a protective environment against desiccation, but also against chemical control measures such as copper and antibiotics. Here, we showed that the SoKa phage significantly reduces biofilm mass for most of its susceptible strains. Therefore, the application of the phage in the epiphytic phase during the autumn and winter seasons could make it possible to prevent biofilm formation, thereby potentially reducing infections during the subsequent endophytic phase.

Although the isolation and characterization of phages has been reported for several plant pathogenic bacteria, results of their application on plants remain scarce. SoKa has shown its potential efficacy in ex planta bioassays on lemon, grapefruit, and orange fruits; significant reductions in symptom development were consequently obtained. However, there is no full reduction of the symptoms in our phage-treated objects, which can likely be attributed to the artificially high bacterial concentrations used in this assay. In addition, we directly injected the bacteria into the fruit, thus taking away the plant host’s primary defense mechanisms and creating the most desirable outcome for Pss, potentially contributing to the high disease incidence. Interestingly, the fruits that were inoculated solely with phage suspensions showed some minor discolorations as well. As we opted to use phage lysates instead of highly purified phage suspensions (e.g., anion exchange chromatography purified phages) in these assays, we hypothesize that bacterial remnants in the lysate such as lipopolysaccharides, flagella, and other pathogen-associated molecular patterns (PAMPs) cause the plant cells to respond to the lysate itself. Nevertheless, this study indicates that phage biocontrol can reduce the symptoms of citrus black pit in fruits, but to increase the efficacy of phages in the field, the persistence of phages in the plant phylloplane should be further improved by the use of protective formulations, the addition of non-pathogenic phage-spreading or adaptive bacterial strains, and the timing and frequency of the application, as previously suggested [8,45,49].

## Data Availability

The sequencing data are available on NCBI GenBank under accession number OM962999.

## References

[1] Xin X.-F., Kvitko B., He S.Y. (2018). *Pseudomonas syringae*: What it Takes to be a Pathogen. Nat. Rev. Microbiol..

[2] Lamichhane J.R., Messéan A., Morris C.E. (2015). Insights into Epidemiology and Control of Diseases of Annual Plants Caused by the *Pseudomonas syringae* Species Complex. J. Gen. Plant Pathol..

[3] Kennelly M.M., Cazorla F.M., de Vicente A., Ramos C., Sundin G.W. (2007). *Pseudomonas syringae* Diseases of Fruit Trees: Progress Toward Understanding and Control. Plant Dis..

[4] Svircev A., Roach D., Castle A. (2018). Framing the Future with Bacteriophages in Agriculture. Viruses.

[5] Holtappels D., Fortuna K., Lavigne R., Wagemans J. (2021). The Future of Phage Biocontrol in Integrated Plant Protection for Sustainable Crop Production. Curr. Opin. Biotechnol..

[6] Sieiro C., Areal-Hermida L., Pichardo-Gallardo Á., Almuiña-González R., de Miguel T., Sánchez S., Sánchez-Pérez Á., Villa T.G. (2020). A Hundred Years of Bacteriophages: Can Phages Replace Antibiotics in Agriculture and Aquaculture?. Antibiotics.

[7] Farooq T., Hussain M.D., Shakeel M.T., Tariqjaveed M., Aslam M.N., Naqvi S.A.H., Amjad R., Tang Y., She X., He Z. (2022). Deploying Viruses against Phytobacteria: Potential Use of Phage Cocktails as a Multifaceted Approach to Combat Resistant Bacterial Plant Pathogens. Viruses.

[8] Jones J.B., Jackson L.E., Balogh B., Obradovic A., Iriarte F.B., Momol M.T. (2007). Bacteriophages for Plant Disease Control. Annu Rev. Phytopathol..

[9] Rombouts S., Volckaert A., Venneman S., Declercq B., Vandenheuvel D., Allonsius C.N., van Malderghem C., Jang H.B., Briers Y., Noben J.P. (2016). Characterization of Novel Bacteriophages for Biocontrol of Bacterial Blight in Leek Caused by *Pseudomonas syringae* pv. *porri*. Front. Microbiol..

[10] Born Y., Bosshard L., Duffy B., Loessner M.J., Fieseler L. (2015). Protection of *Erwinia amylovora* Bacteriophage Y2 from UV-Induced Damage by Natural Compounds. Bacteriophage.

[11] Iriarte F.B., Obradović A., Wernsing M.H., Jackson L.E., Balogh B., Hong J.A., Momol M.T., Jones J.B., Vallad G.E. (2012). Soil-Based Systemic Delivery and Phyllosphere in vivo Propagation of Bacteriophages: Two Possible Strategies for Improving Bacteriophage Persistence for Plant Disease Control. Bacteriophage.

[12] Doron S., Melamed S., Ofir G., Leavitt A., Lopatina A., Keren M., Amitai G., Sorek R. (2018). Systematic Discovery of Antiphage Defense Systems in the Microbial Pangenome. Science.

[13] Ofir G., Sorek R. (2018). Contemporary Phage Biology: From Classic Models to New Insights. Cell.

[14] Tock M.R., Dryden D.T.F. (2005). The Biology of Restriction and Anti-Restriction. Curr. Opin. Microbiol..

[15] Czajkowski R., Ozymko Z., de Jager V., Siwinska J., Smolarska A., Ossowicki A., Narajczyk M., Lojkowska E. (2015). Genomic, Proteomic and Morphological Characterization of Two Novel Broad Host Lytic Bacteriophages ΦPD10.3 and ΦPD23.1 Infecting Pectinolytic *Pectobacterium* spp. and *Dickeya* spp.. PLoS ONE.

[16] Cemen A., Saygili H., Horuz S., Aysan Y. (2018). Potential of Bacteriophages to Control Bacterial Speck of Tomato (*Pseudomonas syringae* pv. *tomato*). Fresenius Environ. Bull..

[17] Quiñones-Aguilar E.E., Reyes-Tena A., Hernández-Montiel L.G., Rincón Enríquez G. (2018). Bacteriófagos En El Control Biológico de *Pseudomonas syringae* pv. *phaseolicola* Agente Causal Del Tizón de Halo Del Frijol. Ecosistemas Recur. Agropecu..

[18] Martino G., Holtappels D., Vallino M., Chiapello M., Turina M., Lavigne R., Wagemans J., Ciuffo M. (2021). Molecular Characterization and Taxonomic Assignment of Three Phage Isolates from a Collection Infecting *Pseudomonas syringae* pv. *actinidiae* and *P. syringae* pv. *phaseolicola* from Northern Italy. Viruses.

[19] Yin Y., Ni P., Deng B., Wang S., Xu W., Wang D. (2019). Isolation and Characterisation of Phages against *Pseudomonas syringae* pv. *actinidiae*. Acta Agric. Scand. Sect. B—Soil Plant Sci..

[20] Pinheiro L.A.M., Pereira C., Esther Barreal M., Pablo Gallego P., Balcao V.M., Almeida A. (2020). Use of Phage Phi 6 to Inactivate *Pseudomonas syringae* pv. *actinidiae* in Kiwifruit Plants: In vitro and ex vivo Experiments. Appl. Microbiol. Biotechnol..

[21] James S.L., Rabiey M., Neuman B.W., Percival G., Jackson R.W. (2020). Isolation, Characterisation and Experimental Evolution of Phage That Infect the Horse Chestnut Tree Pathogen, *Pseudomonas syringae* pv. *aesculi*. Curr. Microbiol..

[22] Rabiey M., Roy S.R., Holtappels D., Franceschetti L., Quilty B.J., Creeth R., Sundin G.W., Wagemans J., Lavigne R., Jackson R.W. (2020). Phage Biocontrol to Combat *Pseudomonas syringae* Pathogens Causing Disease in Cherry. Microb. Biotechnol..

[23] Oueslati M., Mulet M., Zouaoui M., Chandeysson C., Lalucat J., Hajlaoui M.R., Berge O., García-Valdés E., Sadfi-Zouaoui N. (2020). Diversity of Pathogenic *Pseudomonas* Isolated from Citrus in Tunisia. AMB Express..

[24] Berge O., Monteil C.L., Bartoli C., Chandeysson C., Guilbaud C., Sands D.C., Morris C.E. (2014). A User’s Guide to a Database of the Diversity of *Pseudomonas syringae* and Its Application to Classifying Strains in This Phylogenetic Complex. PLoS ONE.

[25] Oueslati M., Mulet M., Gomila M., Berge O., Hajlaoui M.R., Lalucat J., Sadfi-Zouaoui N., García-Valdés E. (2019). New Species of Pathogenic *Pseudomonas* Isolated from Citrus in Tunisia: Proposal of *Pseudomonas kairouanensis* sp. nov. and *Pseudomonas nabeulensis* sp. nov. Syst. Appl. Microbiol..

[26] Adams M.H. (1959). Bacteriophages.

[27] Adriaenssens E.M., van Vaerenbergh J., Vandenheuvel D., Dunon V., Ceyssens P.-J., de Proft M., Kropinski A.M., Noben J.-P., Maes M., Lavigne R. (2012). T4-Related Bacteriophage LIMEstone Isolates for the Control of Soft Rot on Potato Caused by ‘*Dickeya solani*’. PLoS ONE.

[28] Makalatia K., Kakabadze E., Wagemans J., Grdzelishvili N., Bakuradze N., Natroshvili G., Macharashvili N., Sedrakyan A., Arakelova K., Ktsoyan Z. (2020). Characterization of *Salmonella* Isolates from Various Geographical Regions of the Caucasus and Their Susceptibility to Bacteriophages. Viruses.

[29] Altschul S.F., Gish W., Miller W., Myers E.W., Lipman D.J. (1990). Basic Local Alignment Search Tool. J. Mol. Biol..

[30] Nishimura Y., Yoshida T., Kuronishi M., Uehara H., Ogata H., Goto S. (2017). ViPTree: The Viral Proteomic Tree Server. Bioinformatics.

[31] Moraru C., Varsani A., Kropinski A.M. (2020). VIRIDIC—A Novel Tool to Calculate the Intergenomic Similarities of Prokaryote-Infecting Viruses. Viruses.

[32] Brettin T., Davis J.J., Disz T., Edwards R.A., Gerdes S., Olsen G.J., Olson R., Overbeek R., Parrello B., Pusch G.D. (2015). RASTtk: A Modular and Extensible Implementation of the RAST Algorithm for Annotating Batches of Genomes. Sci. Rep..

[33] Tynecki P., Guziński A., Kazimierczak J., Jadczuk M., Dastych J., Onisko A. (2020). PhageAI-Bacteriophage Life Cycle Recognition with Machine Learning and Natural Language Processing 1. bioRXiv.

[34] Joensen K.G., Scheutz F., Lund O., Hasman H., Kaas R.S., Nielsen E.M., Aarestrup F.M. (2014). Real-Time Whole-Genome Sequencing for Routine Typing, Surveillance, and Outbreak Detection of Verotoxigenic *Escherichia coli*. J. Clin. Microbiol..

[35] Seemann T. (2016). ABRicate: Mass Screening of Contigs for Antibiotic Resistance Genes. https://github.com/tseemann/abricate.

[36] Merrill B.D., Ward A.T., Grose J.H., Hope S. (2016). Software-Based Analysis of Bacteriophage Genomes, Physical Ends, and Packaging Strategies. BMC Genom..

[37] Sullivan M.J., Petty N.K., Beatson S.A. (2011). Easyfig: A Genome Comparison Visualizer. Bioinformatics.

[38] Shafique M., Alvi I.A., Abbas Z., Ur Rehman S. (2017). Assessment of Biofilm Removal Capacity of a Broad Host Range Bacteriophage JHP against *Pseudomonas aeruginosa*. APMIS.

[39] Rombouts S., van Vaerenbergh J., Volckaert A., Baeyen S., de Langhe T., Declercq B., Lavigne R., Maes M. (2016). Isolation and Characterization of *Pseudomonas syringae* pv. *porri* from Leek in Flanders. Eur. J. Plant Pathol..

[40] van Overbeek L.S., Nijhuis E.H.M., Koenraadt H., Visser J., van Kruistum G. (2010). The Role of Crop Waste and Soil in *Pseudomonas syringae* pathovar *porri* Infection of Leek (*Allium porrum*). Appl. Soil Ecol..

[41] Magill D.J., Kulakov L.A., Skvortsov T.A. (2020). Genomic Hypervariability of Phage Andromeda Is Unique among Known DsDNA Viruses. bioRxiv.

[42] Sajben-Nagy E., Maróti G., Kredics L., Horváth B., Párducz A., Vágvölgyi C., Manczinger L. (2012). Isolation of New *Pseudomonas tolaasii* Bacteriophages and Genomic Investigation of the Lytic Phage BF7. FEMS Microbiol. Lett..

[43] Husseini A., Akköprü A. (2020). The Possible Mechanisms of Copper Resistance in the Pathogen *Pseudomonas syringae* Pathovars in Stone Fruit Trees. Phytoparasitica.

[44] Compant S., Duffy B., Nowak J., Clément C., Barka E.A. (2005). Use of Plant Growth-Promoting Bacteria for Biocontrol of Plant Diseases: Principles, Mechanisms of Action, and Future Prospects. Appl. Environ. Microbiol..

[45] Iriarte F.B., Balogh B., Momol M.T., Smith L.M., Wilson M., Jones J.B. (2007). Factors Affecting Survival of Bacteriophage on Tomato Leaf Surfaces. Appl. Environ. Microbiol..

[46] Pinheiro L.A.M., Pereira C., Frazão C., Balcão V.M., Almeida A. (2019). Efficiency of Phage Φ6 for Biocontrol of *Pseudomonas syringae* pv. *syringae*: An in vitro Preliminary Study. Microorganisms.

[47] Jones J.B., Svircev A.M., Obradović A.Ž., Harper D., Abedon S., Burrowes B., McConville M. (2021). Crop Use of Bacteriophages. Bacteriophages.

[48] Holtappels D., Fortuna K.J., Moons L., Broeckaert N., Bäcker L.E., Venneman S., Rombouts S., Lippens L., Baeyen S., Pollet S. (2022). The Potential of Bacteriophages to Control *Xanthomonas campestris* pv. *campestris* at Different Stages of Disease Development. Microb. Biotechnol..

[49] Balogh B., Nga N.T.T., Jones J.B. (2018). Relative Level of Bacteriophage Multiplication *in vitro* or in Phyllosphere May Not Predict *in planta* Efficacy for Controlling Bacterial Leaf Spot on Tomato Caused by *Xanthomonas perforans*. Front. Microbiol..

